# Pathogen Reduction in Human Plasma Using an Ultrashort Pulsed Laser

**DOI:** 10.1371/journal.pone.0111673

**Published:** 2014-11-05

**Authors:** Shaw-Wei D. Tsen, David H. Kingsley, Karen Kibler, Bert Jacobs, Sara Sizemore, Sara M. Vaiana, Jeanne Anderson, Kong-Thon Tsen, Samuel Achilefu

**Affiliations:** 1 Department of Radiology, Washington University School of Medicine, St Louis, Missouri, United States of America; 2 U. S. Department of Agriculture, Agricultural Research Service, Food Safety and Intervention Technologies Research Unit, James W. W. Baker Center, Delaware State University, Dover, Delaware, United States of America; 3 Biodesign Institute, Arizona State University, Tempe, Arizona, United States of America; 4 Department of Physics, Arizona State University, Tempe, Arizona, United States of America; 5 Department of Hematology, Barnes Jewish Hospital, St Louis, Missouri, United States of America; 6 Center for Biophysics, Arizona State University, Tempe, Arizona, United States of America; 7 Biochemistry and Molecular Biophysics, Washington University School of Medicine, St Louis, Missouri, United States of America; 8 Biomedical Engineering, Washington University School of Medicine, St Louis, Missouri, United States of America; University of San Francisco, United States of America

## Abstract

Pathogen reduction is a viable approach to ensure the continued safety of the blood supply against emerging pathogens. However, the currently licensed pathogen reduction techniques are ineffective against non-enveloped viruses such as hepatitis A virus, and they introduce chemicals with concerns of side effects which prevent their widespread use. In this report, we demonstrate the inactivation of both enveloped and non-enveloped viruses in human plasma using a novel chemical-free method, a visible ultrashort pulsed laser. We found that laser treatment resulted in 2-log, 1-log, and 3-log reductions in human immunodeficiency virus, hepatitis A virus, and murine cytomegalovirus in human plasma, respectively. Laser-treated plasma showed ≥70% retention for most coagulation factors tested. Furthermore, laser treatment did not alter the structure of a model coagulation factor, fibrinogen. Ultrashort pulsed lasers are a promising new method for chemical-free, broad-spectrum pathogen reduction in human plasma.

## Introduction

Pathogen reduction (PR) is an ideal strategy to combat emerging pathogens and ensure the continued safety of blood products. However, the PR techniques that are currently in use for clinical blood products have limitations that preclude their widespread use (for reviews, see [1]–[3]). The solvent-detergent (SD) method [4], [5], which inactivates enveloped viruses by disrupting lipid membranes, was discontinued in the United States due to an association with unexpected thromboses in some patients [4], [6]. Light-activated photochemicals such as methylene blue [7], [8] and amotosalen [9], [10], which inactivate pathogens through crosslinking, also involve introducing chemicals with concerns of side effects. Such side effects include allergic reactions (in the case of methylene blue) and the generation of antibodies against the treated blood product (in the case of the chemical S-303) [1], as well as the concern of unknown, unpredictable long-term effects that invariably arise with any administered compound. Furthermore, all of the above mentioned methods are ineffective against non-enveloped viruses such as hepatitis A virus (HAV) [6].

Ultrashort pulsed (USP) lasers have recently emerged as an attractive potential technique for pathogen inactivation [11]. The visible USP laser PR technology does not involve potentially toxic or carcinogenic chemicals. Visible USP lasers are non-ionizing and do not covalently modify proteins, thereby reducing concerns of neoantigen formation. Furthermore, USP laser treatment can inactivate a broad spectrum of viruses and bacteria [11]–[14], including non-enveloped viruses that are traditionally difficult to inactivate. Under these treatment conditions, the structure of bovine serum albumin protein was well preserved [14].

In this work we demonstrate inactivation of human immunodeficiency virus (HIV), HAV, and murine cytomegalovirus (MCMV) in human plasma using a USP laser operating at a wavelength of 425 nm. These pathogens are structurally representative of a broad range of viruses including enveloped RNA virus (HIV), non-enveloped RNA virus (HAV), and enveloped DNA virus (MCMV). MCMV serves as a surrogate for human herpesviruses including HCMV. We used MCMV for this study because it is not infectious to humans, allowing us to conduct more detailed studies in a less restrictive environment. MCMV is also readily available in our lab and straightforward to analyze on the bench; more importantly, as an enveloped DNA virus, MCMV serves structurally as a model for clinically relevant enveloped DNA viruses which include Hepatitis B virus, HCMV, and poxviruses. The USP laser technology has been shown to inactivate viruses through mechanical means; in other words, inactivation by the USP laser is most dependent on the structure of a virus.

Furthermore, we assess the function of coagulation factors in treated plasma versus untreated plasma after USP laser irradiation.

## Materials and Methods

### Femtosecond laser irradiation

The excitation source employed in this work was a diode-pumped continuous wave mode-locked Ti-sapphire laser (Trestles-100, Del Mar Photonics, USA). The laser produced a continuous train of 60 fs pulses at a repetition rate of 80 MHz. The output of the second harmonic generation system of the Ti-sapphire laser was used to irradiate the sample. The excitation laser was chosen to operate at a wavelength of *λ*  = 425 nm and with an average power of approximately 120 mW. This laser wavelength was chosen because we have recently demonstrated that it was capable of efficiently inactivating enveloped/non-enveloped, DNA/RNA viruses including HIV, MCMV, murine norovirus, encephalomyocarditis virus, and human papillomavirus [11]. It has a pulse width of full-width at half maximum = 100 fs. A lens was used to focus the laser beam into a spot within the sample volume. Samples were laser-irradiated for 90 min. A magnetic stirring device was used to facilitate exposure of the sample to the laser beam. Irradiation was carried out at 22°C and with the single laser beam excitation. After laser irradiation, samples were immediately stored at −80°C.

### Viruses and infectivity assays

#### HIV inactivation

HIV stock was propagated in MT-4 cells following transfection of HeLa cells with pNL4-3 plasmid (both MT-4 cells an pNL4-3 were kind gifts of Dr. Kuan-Teh Jeang). Approximately 4×10^6^ reverse transcriptase (RT) units of NL4-3 stock was added to pooled normal plasma (George King Biomedical, Inc., Overland Park, KS). The infected plasma was placed into each of 6 glass vials for transport to the laser lab. Each of the 3 vials containing a stir bar was laser-irradiated as described above. During the treatment time, a vial serving as a room temperature control was placed in a beaker in the same room. During each treatment time, 4 of the 6 vials remained on ice. When each of 3 vials had been irradiated, all 6 vials were transported back to the HIV lab, where they were stored at −80°C until used in a Multinuclear Activation Galactosidase Indicator (MAGI) assay. For the MAGI assay, the MAGI cells (U373-MAGI-CXCR4 CEM, catalog #3596, contributed by Dr. Michael Emerman) were obtained through the NIH AIDS Research and Reference Reagent Program, Division of AIDS, NIAID, NIH, Bethesda, USA. The standard MAGI assay protocol was followed. Briefly, plasma/virus mixture was added to MAGI cells, incubated for 48 h, fixed with 4% paraformaldehyde for 2 h, and X-gal (Teknova, USA, Cat # X1205) staining was visualized. Three irradiated samples, three room temperature control samples, and the original virus stock were used in the assay. The final dilution factor for treated samples and controls was 1∶2 compared to the original virus stock dilution used in the assay.

#### HAV inactivation

HAV stock was obtained from the American Type Culture Collection (Manassas, VA) as VR1402, a cell culture-adapted cytopathic clone of strain HM-175 that was originally designated as HM-175/18f. The virus was propagated on fetal rhesus monkey kidney (FRhK-4) cells as previously described [15]. The HAV stock was stored at −70°C in DMEM (Gibco, Grand Island NY) with 10% fetal bovine serum (FBS) (Gibco) prior to use. To partially purify HAV, the virus was pelleted at 490,000×g for 6 h, followed by resuspension of the HAV in human plasma (George King Biomedical Inc., Overland Park, KS) and filtration through a 0.1 µm filter. Samples of human plasma alone or human plasma spiked with HAV were laser-irradiated as described above. Plaque assay was performed by making an initial 100-fold dilution followed by 10-fold serial dilutions made in Earle’s balanced salt solution (Gibco) and infecting 100-mm dishes confluent with FRhK-4 cell and infecting with 2 ml of virus dilution as described previously [16]. After 2 h, the plates were overlaid with DMEM medium with 5% FBS, and 1% agarose (Sigma-Aldrich, St. Louis, MO). At 17 days post-inoculation, HAV was inactivated by 10% formaldehyde treatment, the agarose overlay was removed, and HAV plaques were visualized by crystal violet staining (Fisher Scientific, Kalamazoo, MI).

#### MCMV inactivation

Murine embryonic fibroblast 10.1 (MEF 10.1) cells [17] (a generous gift from Dong Yu, Washington University School of Medicine, St Louis, MO) were cultured in Dulbecco’s Modified Eagle Medium (DMEM), supplemented with 10% FBS, 1 mM sodium pyruvate, and nonessential amino acids. GFP-expressing MCMV virus (hereafter referred to as “MCMV”) was generated as previously described [18]. To produce viral stocks, MEF 10.1 cells were infected with MCMV at a low multiplicity of infection. Cell supernatants were harvested 24 h post-infection after 100% cytopathic effect and cleared of cell debris by centrifugation. Extracellular virions were pelleted by ultracentrifugation with sorbitol cushion and resuspended in phosphate-buffered saline (PBS). Samples of human plasma alone or human plasma spiked with MCMV were laser-irradiated as described above. Viral titers were determined using a median tissue culture infectious dose (TCID_50_) assay, as previously described [13]. Briefly, MEF 10.1 cells were seeded into 96-well plates at a density of 1.25×10^5^ cells/ml and incubated overnight. Cells were approximately 80% confluent at the time of infection. Laser-treated or control (untreated) viruses were serially diluted and added to cells, which were incubated for 4 days. Viral titers were determined on day 4 post-infection by scoring each well for GFP-positive cells using a fluorescent microscope.

### Coagulation factor assays

To evaluate the activity of coagulation factors in plasma with or without USP laser treatment, we performed standard coagulation factor assays [19]. Plasma samples derived from the same lot of pooled normal human plasma (George King Biomedical, Inc.) were kept untreated or laser-treated as described previously. Factor (F) II, FV, FVII, FVIII, FIX, FX, FXI, FXII, and fibrinogen were evaluated in Barnes-Jewish Hospital Laboratory, St. Louis, MO. Assays were completed on the IL ACL TOP 700, according to the manufacturer’s protocols contained within the package inserts (Instrumentation Laboratories Company, Bedford, MA). Plasma levels of FII, FV, FVII, and FX were determined using functional assays based on the prothrombin time with human plasma immunodepleted of FII, FV, FVII, or FX. Similarly, plasma levels of FVIII, FIX, FXI, and FXII were determined using the activated partial thromboplastin time with human plasma immunodepleted of FVIII, FIX, FXI, and FXII. Plasma deficient in FII, FV, FVII, FVIII, FIX, FX, FXI, or FXII consisted of human plasma that was immunodepleted of the individual coagulation factors, and were purchased from Instrumentation Laboratories Company. HemosIL RecombiPlasTin 2G and HemosIL SynthASil (Instrumentation Laboratories Company) were additional reagents used in the assay. Fibrinogen level was determined by a quantitative assay based on the Clauss method [20], [21] using Q.F.A. Thrombin (Bovine) reagent (Instrumentation Laboratories Company).

### Protein gel electrophoresis

To evaluate protein aggregation of samples with or without USP laser treatment, we employed gel electrophoresis analysis. For SDS-PAGE, control (untreated) or laser-treated samples containing equivalent quantities of plasma were boiled in loading buffer (dH_2_O (47.5%), 0.5 M Tris pH 6.8 (12.5%), glycerol (10%), SDS (20%), β-mercaptoethanol (5%), and bromophenol blue (5%)) under reducing conditions, and separated on a Mini-Protean TGX 10% precast polyacrylamide gel (Bio-Rad, Hercules, CA). For native PAGE, control (untreated) or laser-treated samples containing equivalent quantities of plasma were separated on the 10% gel under non-denaturing conditions. Protein bands were visualized with Coomassie blue staining (LabSafe Gel Blue, G-Biosciences).

### Fibrinogen protein preparation

Purified human fibrinogen was obtained from Haemtech Technologies, Inc (Essex Junction, VT). For protein structure characterization measurements, fibrinogen solutions (2.5 mg/ml) were prepared by dissolving pure, lyophilized peptide in PBS buffer which was filtered with a 0.02 µm Whatman Anotop25 filter. The actual concentration was checked by measuring the absorption of the sample with a Cary50 UV-Vis spectrophotometer (Agilent, Inc., Santa Clara, CA), using an extinction coefficient of fibrinogen at 280 nm of 5.12×10^5^ M^−1^ cm^−1^. Dynamic light scattering (DLS), circular dichroism (CD) and absorbance measurements were carried out immediately before and after irradiation of the sample.

### Dynamic light scattering measurements

To determine the effect of USP laser treatment on the aggregation of fibrinogen protein, we employed dynamic light scattering analysis. Before and after irradiation, fibrinogen samples were centrifuged at 12,000×g for 15 min to clear them from dust, and 5 µl aliquots were taken to measure the DLS signal of the sample. Autocorrelation functions of the scattered intensity at 90° scattering angle were collected at 25°C with a 5 sec acquisition time, using a Wyatt Technology DynaPro NanoStar with a 658 nm, 120 mW GaAs linearly polarized laser. Measurements of fibrinogen were done in a 1 µl MicroCuvette (Wyatt Technology Corp., Santa Barbara, CA), previously calibrated with clear water. Data were analyzed using Wyatt Technology Dynamics 7 software, using a regularization fit method to determine hydrodynamic radii of fibrinogen from the autocorrelation functions.

### Circular dichroism measurements

To examine laser-induced alterations in the secondary structure of fibrinogen protein, circular dichroism measurements were made. Immediately before and after irradiation, an aliquot of the fibrinogen sample was diluted (1∶20 for the 2.5 mg/ml sample and 1∶10 for 1.5 mg/ml and 0.6 mg/ml samples) and transferred to a 1 mm quartz cuvette (Starna Cells, Atascadero, CA, USA) and circular dichroism spectra were measured at room temperature using J-710 spectropolarimeter (Jasco Instruments, Easton, MD). Far UV spectra from 200 nm to 250 nm were obtained by averaging over eight scans, with a 1 nm bandwidth, 0.5 nm pitch and 50 nm/min scan speed, and then buffer subtracted. Fibrinogen concentration was estimated from the absorption spectra of undiluted samples, measured before and after laser irradiation. These values were used to convert CD signals to mean residue ellipticity (MRE). We note that a slight decrease in the signal amplitude was observed each time the samples were transferred from one container to another. This was consistent with the expected loss of protein due to adsorption of fibrinogen to the surface of containers [22].

### Statistics

Quantitative experiments were performed in triplicate. Differences between mean titers of control and laser-treated virus were analyzed by Student’s t-test using Graphpad Prism and Microsoft Excel software. p<0.05 was used as a threshold for statistical significance.

## Results

### USP laser treatment inactivates viruses in human plasma

**Figure 1:**
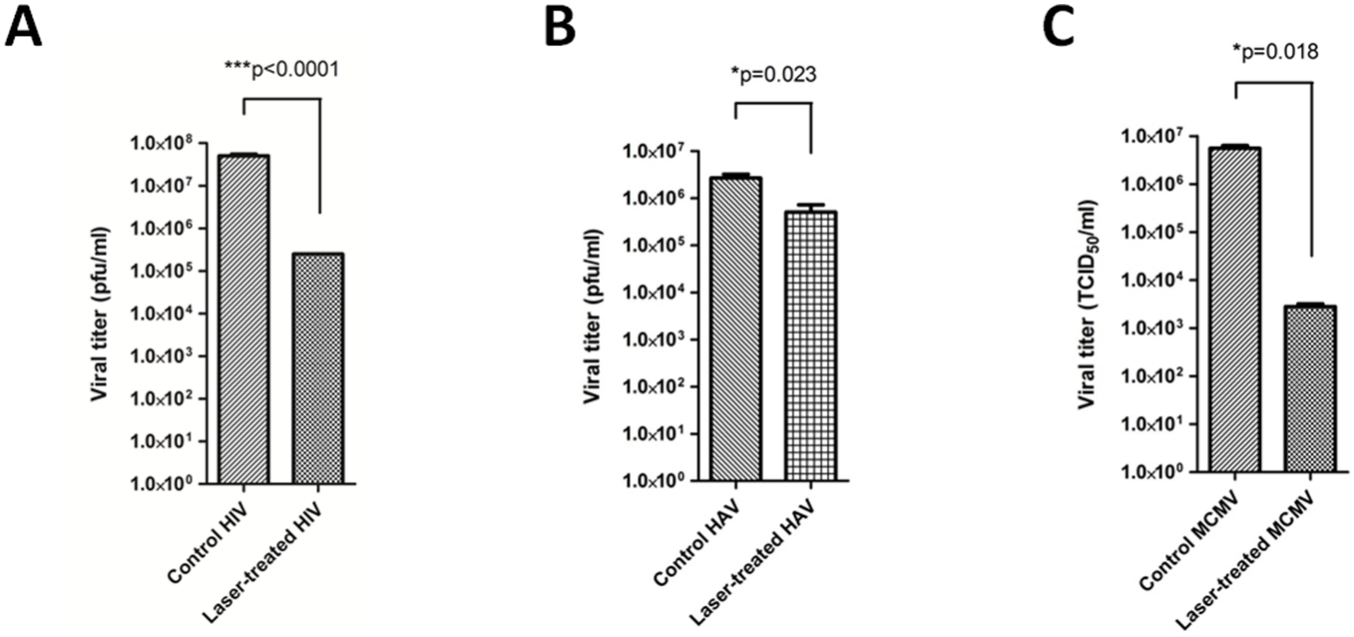
Inactivation of viruses in plasma using a USP laser. Human plasma containing HIV (A), HAV (B), or MCMV (C) were treated with the USP laser. For the HIV-spiked plasma, viral titer was assessed by plaque assay in MAGI cells. For the HAV-spiked plasma, viral titer was assessed by plaque assay in fetal rhesus monkey kidney cells. For the MCMV-spiked plasma, viral titer was assessed by TCID_50_ assay in murine embryonic fibroblast cells. Results are representative of triplicate experiments and are shown as means ± SEM.

For this study, we chose HIV and HAV as medically significant enveloped and non-enveloped RNA viruses, respectively, and we chose MCMV as a representative enveloped DNA virus whose results could be extrapolated to relevant human pathogens such as cytomegalovirus and hepatitis B virus. To demonstrate that the USP laser treatment can inactivate viruses in plasma, aliquots of HIV, HAV, or MCMV were spiked into human plasma and treated with the laser. USP laser treatment of virus-spiked plasma samples resulted in approximately 2-log, 1-log, and 3-log reductions in HIV, HAV, and MCMV titers, respectively (Figure 1A–C). The reduction in HAV titers after USP laser treatment exceeds that achieved by the currently licensed SD and amotosalen techniques [6]. It is anticipated that further optimization of laser parameters such as wavelength (i.e., operating at wavelengths where absorption by bilirubin/hemoglobin are minimal) would yield greater inactivation of viruses. These data indicate that USP laser treatment can achieve clinically meaningful reduction of viruses in human plasma.

### The function of human plasma proteins is preserved after USP laser treatment

To determine the functional integrity of human plasma after USP laser treatment, we laser-treated human plasma alone using the same conditions used to inactivate the viruses. After treatment, samples were subjected to standard coagulation assays. Human coagulation factors in laser-treated plasma showed preservation of activity comparable to that achieved with other clinically tested PR methods [6]. The percent retention of individual coagulation factors after laser treatment relative to control is shown in Table 1. With the exception of Factor VII, the activities of all the plasma proteins were near or within the normal reference range. Relative to non-irradiated sample, Factors II, V, VII, IX, and X showed ≥90% retention. Factor XII and fibrinogen showed ≥70% retention. Factors VIII and XI were the most sensitive and showed 68% and 54% retention, respectively. Although Factor II activity was increased by 20% relative to control (p = 0.04), the value still fell within the normal reference range, and similar small increases in the activity of certain factors after treatment using current PR methods have been reported, potentially due to the intrinsic variation in the assay^2^. As an example, riboflavin treatment was previously shown to increase the measured activity of Factor XIII to 113% the control value [6]. Therefore, we did not attribute clinical significance to the observed increase in Factor II activity. Interestingly, the USP laser treatment caused a dramatic enhancement in the measured activity of Factor VII in plasma (Table 1). This effect could either be direct (i.e., laser-induced structural changes in Factor VII protein) or indirect (i.e., laser-induced damage to factor(s) that inhibit Factor VII activity). We believe that the latter scenario is more likely, since it is difficult to envision how USP laser treatment could directly increase the activity of Factor VII protein by altering its native structure. Therefore, a plausible explanation is that the USP laser treatment may cause damage to Factor VII inhibitor(s) that are present in human plasma (such as tissue factor pathway inhibitor, TFPI), which leads to an apparent “enhancement” in measured Factor VII activity. It is expected that further optimization of laser parameters such as wavelength (i.e., operating at wavelengths where absorption by bilirubin and/or hemoglobin are minimal) would yield greater preservation of plasma proteins. These data demonstrate that USP laser irradiation retains coagulation factor activities in an acceptable range for clinical translation.

**Table 1:** Retention of coagulation factor activity for USP laser-treated plasma.

Coagulation factor	Control plasma	Laser-irradiated plasma	Normal reference range	% retention after treatment
Fibrinogen (mg/dl)	218±4	158.5±2.5	170–400	73
Factor II (IU/ml)	0.82±0.02	0.98±0.03	0.75–1.30	120
Factor V (IU/ml)	0.84±0.01	0.80±0.06	0.50–1.25	95
Factor VII (IU/ml)	0.92±0.01	2.22±0.23	0.50–1.75	241
Factor VIII (IU/ml)	0.72±0.01	0.49±0.01	0.50–1.60	68
Factor IX (IU/ml)	1.01±0.01	0.96±0.09	0.55–1.60	95
Factor X (IU/ml)	1.02±0.03	1.04±0.03	0.60–1.60	102
Factor XI (IU/ml)	1.01±0.01	0.55±0.09	0.60–1.40	54
Factor XII (IU/ml)	0.90±0.00	0.69±0.02	0.45–1.70	77

### USP laser treatment does not induce detergent-resistant aggregation in human plasma proteins

**Figure 2:**
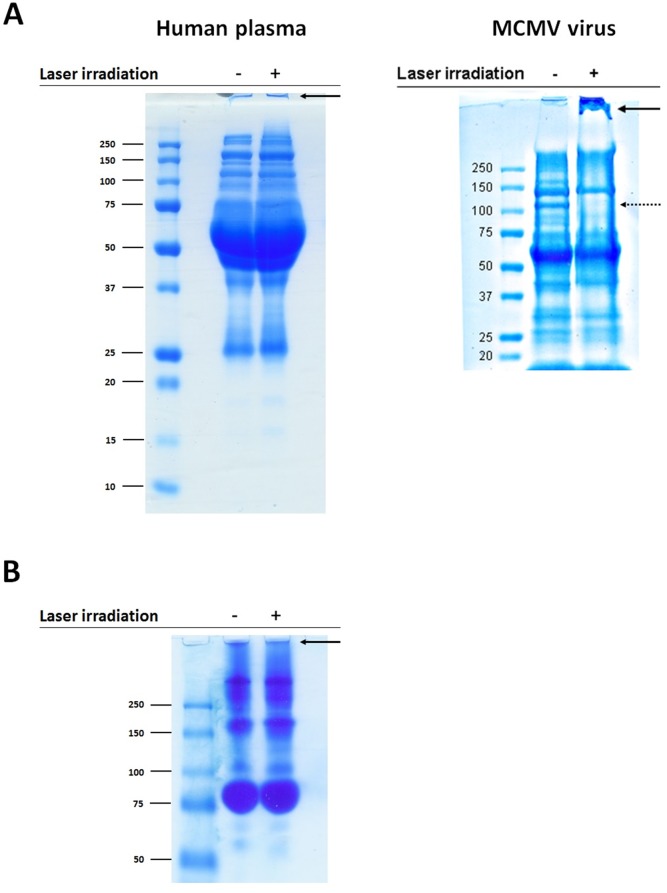
SDS-PAGE and native PAGE analysis of control and USP laser-treated plasma proteins. (A) On the left is shown the SDS-PAGE of control and laser-treated plasma; on the right, for comparison, is shown the SDS-PAGE of laser-treated MCMV virus adapted from Tsen *et al* [13] (reprinted with permission from the Society of Photo-Optical Instrumentation Engineers). Control (untreated) or USP laser-treated plasma were boiled in reducing buffer and separated on a 10% gel. Proteins were visualized by Coomassie blue stain. The solid arrow indicates the location of low mobility detergent-resistant aggregates; the dotted arrow indicates missing band(s) corresponding to aggregated proteins. (B) Native PAGE of control and laser-treated plasma. Control (untreated) or USP laser-treated plasma were separated on a 10% gel. Proteins were visualized by Coomassie blue stain. Arrows indicate location of low mobility detergent-resistant aggregates.

To determine the effects of laser treatment on human plasma proteins, we analyzed control (untreated) and laser-treated plasma by SDS-PAGE (Figure 2A, left panel). Both control and laser-treated plasma contained some intrinsic level of detergent-resistant aggregates as evidenced by the presence of low-mobility protein complexes that are unable to migrate through the gel. However, USP laser treatment of plasma did not cause any significant qualitative increase in these aggregates. We were also unable to find evidence for any laser-induced increase in detergent-resistant aggregates in plasma by native PAGE (Figure 2B). These data suggest that USP laser treatment does not induce detergent-resistant aggregation among plasma proteins.

### The secondary structure and aggregation state of purified human fibrinogen are unaltered after USP laser treatment

As shown in Table 1, the function of some coagulation factors was reduced after laser treatment. In particular, the function of fibrinogen, which is the coagulation factor that occurs at the highest concentration in plasma (∼2–4 mg/ml) among the factors tested, was reduced to 73% of control. To determine if direct, laser-induced change in protein structure and/or aggregation state of the protein itself was responsible for this reduction in function, we analyzed the structure and aggregation state of purified human fibrinogen protein at physiological concentration (2.5 mg/ml) after USP laser treatment using circular dichroism and dynamic light scattering analysis, respectively.

**Figure 3:**
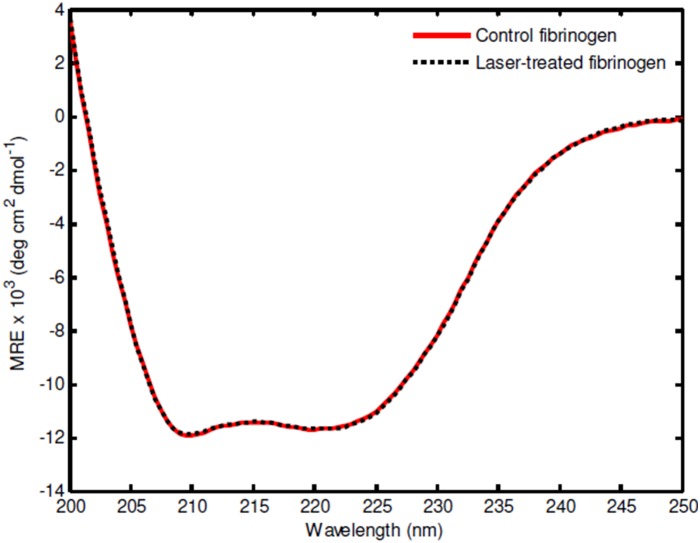
Structural analysis of control and laser-treated fibrinogen protein. Control (untreated) or laser-treated fibrinogen protein was analyzed by circular dichroism. The red line indicates the CD spectrum of control fibrinogen, while the dotted black line indicates laser treated fibrinogen. The spectra (in Mean Residue Ellipticity, rescaled for concentration) show virtually complete overlap.

We found no evidence for laser-induced alterations in the secondary structure of fibrinogen by circular dichroism measurements (Figure 3). Furthermore, we did not observe any significant laser-induced aggregation of fibrinogen protein by dynamic light scattering, as evidenced by near-identical proportions of monomer in both control and laser-treated groups (Table 2). We note that these values represent the percentage of scattered intensity from monomers (i.e. species with hydrodynamic radius of 12 nm). In both the controls and the irradiated samples, the remaining 40% of intensity came from a much larger species, with a hydrodynamic radius around 100 nm. Because large aggregates scatter much more than monomers, these values indicate that roughly 90% of the protein mass was monomeric (assuming that the aggregates are spherical). Similar results were obtained with fibrinogen at lower concentrations (1.5 mg/ml and 0.6 mg/ml). These findings indicate that USP laser treatment does not directly cause alterations in the secondary structure or aggregation state of purified fibrinogen protein itself when the protein is treated at physiological or sub-physiological concentrations.

**Table 2:** Dynamic light scattering measurements on the aggregation state of fibrinogen.

Fibrinogen concentration	Sample	% monomer
2.5 mg/ml	Control fibrinogen	60+/−3
	Laser-treated fibrinogen	61+/−7
1.5 mg/ml	Control fibrinogen	63+/−4
	Laser-treated fibrinogen	56+/−9
0.6 mg/ml	Control fibrinogen	65+/−7
	Laser-treated fibrinogen	57+/−10

## Discussion

In this report we demonstrate inactivation of representative viruses in plasma using a visible USP laser, with preservation of plasma proteins. Although we achieved significant reductions in virus, we note that the laser inactivation efficiency for viruses in plasma is decreased relative to that for viruses in phosphate-buffered saline (PBS) [13], [23]. This is likely due to the reduced penetration of light through plasma, which is less transparent than PBS. This can be remedied by designing a laser treatment chamber that is sufficiently thin (<1 mm depth) to maximize light transmission. For example, in a continuous-flow treatment scenario (for example using a syringe pump), a tube or capillary with a small depth would ensure more complete exposure of the sample to the laser light. In future studies, we estimate that the use of more powerful commercially available lasers would enable focused spot sizes on the order of millimeters. Coupled with continuous flow systems, this could provide a plasma treatment efficiency on the order of ∼100 ml per minute which would bring the technology closer to clinical plausibility.

After USP laser treatment, most coagulation factor activities were retained in the range of 70–100% control. Typically, in the field of blood transfusion the acceptable retention percentage of protein activities for PR methods in human plasma is about 70% [6]. For example, methylene blue (MB)-treated plasma has been used extensively in the clinic, and this method reduces the activity of several proteins to this threshold (fibrinogen, 65%; FVIII, 67%) [6]. Other clinically employed methods such as amotosalen have similar damage profiles. The proteins (except Factor XI) in human plasma treated with our USP laser technology were retained at around 70% activity or above (Table 1); therefore, it should be considered to be meeting this requirement. We believe that this non-invasive USP laser technology will have minimal side effects partly because of the nature of the inactivation mechanism by the USP laser irradiation, and partly because no chemicals are introduced in the treatment process. Therefore, using human plasma treated with the USP laser irradiation would be safer compared with other PR methods. Although the level of preservation is in the range of other established PR methods, there is potential for improvement. Human plasma contains bilirubin, a molecule that absorbs light at the 425 nm wavelength we used for laser PR [24]. This absorption leads to intermolecular energy transfers that may negatively affect the structure of bilirubin-associated proteins in plasma. Since viruses in PBS alone are efficiently inactivated by USP laser treatment [12]–[14], [23], [25]–[27], bilirubin is clearly not required for laser PR. Thus it is possible that by selecting a different wavelength where absorption by bilirubin is minimized, the detrimental effects of the USP laser treatment on plasma proteins can be mitigated. For example, use of near-infrared excitation wavelengths above 700 nm will improve the penetration depth of light and minimize absorption of light by plasma proteins for effective pathogen inactivation.

Previous data supported a model whereby the USP laser inactivates enveloped viruses such as MCMV by laser-driven excitation of vibrational modes within viral capsids, resulting in aggregation of densely-packed tegument and capsid proteins [13], [28]. The laser treatment caused the formation of large, strongly bound aggregates of viral capsid/matrix proteins that did not readily dissociate under denaturing or reducing conditions, which we term “detergent-resistant aggregates” [13]. Figs. 2A and 2B demonstrate that USP laser treatment does not induce detergent-resistant aggregates in human plasma. On the other hand, our previous gel results on the control and USP laser-treated MCMV in buffered solution, which is shown in the right panel of Fig. 2A, indicated that USP laser irradiation caused detergent-resistant aggregates, leading to the inactivation of MCMV.

Ideally, it is the gel analysis for the MCMV in human plasma that needs to be made. However, we have found that the signature signal of MCMV was overwhelmed by the tremendously large background contributions from the various components present in the human plasma in the gel analysis of the MCMV spiked in human plasma. As a result, the analysis is too complicated to be reliably made. Therefore, we chose to analyze the best possible experiments next to the ideal one, namely purified MCMV in buffered solution. Because the analysis of the gel results on the purified MCMV is not the ideal one, as a compromise, we make a reasonable assumption that the effects of USP laser treatment on enveloped viruses such as MCMV are the same in the buffered solution as in the human plasma. This assumption is reasonable because it is supported by our observations that MCMV is efficiently inactivated by the USP laser irradiation both in the buffered solution and in human plasma. Under this assumption, it can be shown that the protein aggregation induced by the USP laser irradiation is a plausible mechanism for our experimental observations that the USP laser efficiently inactivates viruses while retaining protein activities in the human plasma. The protein aggregation induced by the USP laser irradiation has been demonstrated to be density-dependent [13]; namely, the more closely the proteins are packed, the larger the probability of protein aggregation. Because the protein density is significantly higher within the MCMV virion than in human plasma, it is expected that protein aggregation by the USP laser irradiation would be much more probable within the MCMV than in the human plasma, leading to the efficient inactivation of MCMV but retention of the majority of coagulation factor activities in human plasma.

We also note that that ideally, it is the fibrinogen within human plasma that needs to be analyzed. However, we have found that the CD and DLS analyses for fibrinogen in human plasma are too complicated to be reliably made due to the presence of background contributions from other components present in the human plasma. Therefore, we chose to analyze the best possible experiments next to the ideal one, namely the CD and DLS of purified fibrinogen. Because the CD and DLS analysis of purified fibrinogen is not the ideal one, as a compromise, we choose not to make a definitive conclusion based on the CD and DLS results on purified fibrinogen. Instead, we conclude that the CD and DLS results on purified fibrinogen provide a possible clue to explain why the USP laser irradiation preserves the majority (73%) of the fibrinogen activity in human plasma.

We were unable to correlate the observed reduction in plasma protein function with any changes in the secondary structure or aggregation state of the proteins. There are two potential explanations for this observation. Firstly, the USP laser may cause only minimal structural damage to these proteins at a level undetectable by circular dichroism, dynamic light scattering, and SDS-PAGE. In this case, more sensitive methods such as nuclear magnetic resonance may be required to elucidate these changes. Alternatively, the function of plasma proteins might be indirectly inhibited by laser-induced damage to other factors in plasma that promote the coagulation activity of these proteins. Indeed, the absorption of laser light by other molecule(s) present in plasma, such as bilirubin, might lead to energy transfer from these molecule(s) to closely bound/closely associated proteins. Studies on the effect of bilirubin on plasma protein preservation after USP laser treatment are currently underway.

Visible USP laser treatment provides important advantages in the field of PR. The USP laser technology does not involve introducing potentially toxic or carcinogenic chemicals, and thus avoids possible side effects from such additives. Unlike the SD and amotosalen methods, which have seen significant clinical use in Europe, USP laser treatment can inactivate non-enveloped viruses. In addition, USP lasers are non-ionizing and do not disrupt covalent bonds, thereby reducing the potential to generate immunogenic neoantigens in blood products. Furthermore, USP lasers are environmentally friendly, circumventing the need to incorporate undesirable compounds such as mercury which is used in UV lamps.

Such qualities make USP lasers a promising potential method for sterilization not only of blood products, but of pharmaceuticals, biologicals, and cell culture as well. As described above, the ISRS inactivation mechanism of USP lasers is very likely to leave small molecule pharmaceuticals and soluble recombinant proteins structurally undamaged. In addition, we have previously shown that a therapeutic window exists for which viruses and bacteria can be inactivated without causing death in human cells [12]. Studies are currently ongoing to conclusively demonstrate these applications.

## Conclusion

We have performed PR experiments on three representative viruses in human plasma by using a visible USP laser. Our experimental results demonstrate that it is feasible to use this novel USP laser technology to inactivate viruses while retaining the function of coagulation factors in human plasma at a clinically acceptable level. This is the first proof-of-concept study of PR in human plasma using USP lasers. This chemical-free USP laser technology has potential advantages over current PR techniques.
